# The Association Between Periodontal Inflamed Surface Area (PISA), Inflammatory Biomarkers, and Mitochondrial DNA Copy Number

**DOI:** 10.3390/jcm14010024

**Published:** 2024-12-25

**Authors:** Romana Mance Kristan, Staša Jurgec, Uroš Potočnik, Marko Marhl, Rok Gašperšič

**Affiliations:** 1 Community Health Centre dr. Adolf Drolc Maribor, Ulica talcev 9, 2000 Maribor, Slovenia; 2 Department of Periodontology, Faculty of Medicine, University of Maribor, Taborska ulica 8, 2000 Maribor, Slovenia; 3 Centre for Human Genetics & Pharmacogenomics, Faculty of Medicine, University of Maribor, Taborska ulica 8, 2000 Maribor, Slovenia; 4 Laboratory for Biochemistry, Molecular Biology & Genomics, Faculty of Chemistry and Chemical Engineering, University of Maribor, Smetanova ulica 17, 2000 Maribor, Slovenia; 5 Department for Science and Research, University Medical Centre Maribor, Ljubljanska ulica 5, 2000 Maribor, Slovenia; 6 Department of Biophysics, Faculty of Medicine, University of Maribor, Taborska ulica 8, 2000 Maribor, Slovenia; 7 Faculty of Education, University of Maribor, Koroška cesta 160, 2000 Maribor, Slovenia; 8 Faculty of Natural Sciences and Mathematics, University of Maribor, Koroška cesta 160, 2000 Maribor, Slovenia; 9 Department of Oral Medicine and Periodontology, Faculty of Medicine, University of Ljubljana, Hrvatski trg 6, 1000 Ljubljana, Slovenia

**Keywords:** PISA, mitochondrial DNA copy number, mtDNA buccal swab, oxidative biomarkers

## Abstract

**Background/Objectives**: Periodontitis is an inflammatory disease induced by bacteria in dental plaque that can activate the host’s immune-inflammatory response and invade the bloodstream. We hypothesized that a higher periodontal inflamed surface area (PISA) is associated with higher levels of inflammatory biomarkers, lower levels of antioxidants, and mitochondrial DNA copy number (mtDNAcn). **Methods**: Using periodontal parameters, we calculated the PISA score, measured the levels of inflammatory biomarkers and antioxidants in the serum, and took buccal swabs for mtDNA and nuclear DNA (nDNA) extraction. **Results**: Higher PISA was associated with higher CRP levels, higher leukocyte, neutrophil, and erythrocyte counts, and lower magnesium-to-calcium ratio, but not with mtDNAcn. A higher number of deep pockets was associated with higher leukocytes and neutrophil counts and higher uric acid levels. **Conclusions**: The PISA score might be an appropriate parameter to assess the inflammatory burden of periodontitis, but not to assess mitochondrial dysfunction after mtDNA isolation from buccal swabs.

## 1. Introduction

Periodontitis is a chronic, multifactorial inflammatory disease associated with dysbiotic plaque biofilms [1] as the primary risk factor for periodontitis, but the host inflammatory immune response plays an equally important role in the development and progression of the disease. The persistent inflammation of periodontal tissues leads to clinical attachment loss (CAL), alveolar bone loss, and periodontal pocket formation associated with bleeding on probing (BOP) because of ulcerated pocket epithelium [2], which becomes permeable to bacteria and their products. The size of this area, referred to as the periodontal inflamed surface area (PISA), can be estimated by calculating the probing depths and BOP [3,4]. After invading the periodontal tissues, bacteria and their products can enter the systemic circulation [5], activating the host’s inflammatory and immune response and, thus, affecting the patient’s health [1].

The impact of periodontitis on systemic health can be assessed by biomarkers such as C-reactive protein, leukocyte counts, especially neutrophils, red blood cell count, haemoglobin, and various cytokines levels measured in the peripheral blood. White blood cell counts, especially neutrophils [6,7,8] and C-reactive protein (CRP), are elevated in periodontitis [6,9,10,11,12], but the relationship between periodontitis and red blood cell count is inconsistent [13,14,15]. Furthermore, studies show that higher Mg/Ca levels are associated with a lower risk of periodontitis [16,17], while periodontitis is closely associated with oxidative stress, which plays an important role in the development and progression of the disease. Oxidative stress refers to an imbalance between the production of reactive oxygen species (ROS) and the body’s antioxidant defence mechanisms [18]. Antioxidants are important to prevent tissue damage caused by an overproduction of ROS [19]. One of the most important antioxidants is uric acid. The increase of uric acid levels is related to the severity of periodontitis, suggesting that uric acid is not only an antioxidant, but also proinflammatory [20]. It is also possible that elevated serum uric acid levels have a protective effect against periodontitis [21]. The other important antioxidant is coenzyme Q10 [22], as its deficiency has been observed in the gingival tissue of periodontal patients [23], and the use of CoQ10 as an adjuvant to non-surgical therapy showed a positive clinical impact [24,25,26].

Mitochondrial DNA copy number (mtDNAcn) has been shown to reflect the ratio between mitochondrial and nuclear DNA copy number [27], and oxidative stress is known to affect this number, which can serve as a biomarker of mitochondrial health. Decreased mtDNAcn may indicate mitochondrial stress or damage, as commonly seen in inflammatory diseases such as periodontitis [28]. MtDNA is more susceptible to oxidative damage than nuclear DNA (nDNA) [29,30,31]. The number of mitochondria in a cell varies depending on the type of tissue, which is due to the different energy requirements of each tissue. Therefore, the mtDNA/nDNA ratio can be used as an estimate of the variability in the number of cells and their mitochondrial content between samples. Since each cell contains a single nucleus, but multiple mitochondria, the ratio normalizes the mtDNAcn to the number of cells present [32,33]. This is particularly important when analysing samples with different cell compositions, such as buccal swabs containing a mixture of epithelial cells and leukocytes [34]. Many studies show that changes in mtDNAcn are associated with systemic diseases such as diabetes, cardiovascular diseases, various types of cancer, cognitive impairment, and neurodegenerative diseases [30,35,36,37,38,39,40]. In patients with periodontitis, the expected ratio of mtDNA to nDNA may vary due to mitochondrial dysfunction and abnormalities associated with the disease [28]. At different stages of periodontitis, the expected ratio of mtDNA to nDNA may vary due to disease progression and associated mitochondrial dysfunction [41].

Previous studies have already demonstrated a bidirectional relationship between periodontal parameters and several of the above-mentioned systemic parameters related to inflammatory responses (e.g., CRP), oxidative stress (e.g., coenzyme Q10), metabolic parameters (e.g., HbA1c), and mitochondrial dysfunction (e.g., changes in mtDNAcn) [13,17,28,42,43]. To determine mitochondrial dysfunction in the context of other systemic diseases or conditions, the buccal swab has mainly been used and proven to be a suitable method due to its simplicity and non-invasiveness [44,45]. To our knowledge, the swab has not yet been used for the extraction of mtDNA in the context of periodontitis. Our aim was to test whether a buccal swab could serve as an effective source of mtDNA and nDNA, and to determine the relationship between the number of mtDNAcn from the buccal swab and the PISA score or number of deep pockets. We hypothesised that mtDNAcn is associated with a higher PISA score, higher levels of inflammatory biomarkers, and lower levels of antioxidants, indicating mitochondrial damage, higher inflammatory burden, and higher oxidative stress in patients with periodontitis. For these reasons, we exclusively collected epithelial cells with a buccal swab at or near the site of inflammation, rather than peripheral blood mononuclear cells, to isolate mtDNA and nDNA.

## 2. Materials and Methods

### 2.1. Study Population

The study was conducted on patients referred for periodontal treatment at the Municipal Health Centre in Maribor, Slovenia. A health questionnaire and an interview with the patients were used to collect general and socioeconomic data, medical history, information on medication, antibiotic treatment, and smoking. Pregnant and breastfeeding mothers, patients with systemic diseases that affect the inflammatory and immune response of the body, such as cancer and autoimmune diseases, patients taking medications that affect the gums (e.g., calcium channel blockers or antiepileptic drugs), patients who had taken antibiotics or anti-inflammatory drugs in the last six months, smokers, and anyone who had previously been treated for periodontitis were excluded from our study. Before inclusion in the study, each patient underwent a clinical examination. Patients with fewer than 20 teeth were excluded from our study. Oral hygiene was assessed by measuring the plaque index, inflammation by BOP, and periodontal tissue involvement by measuring pocket probing depth (PPD), gingival recession (REC), and clinical attachment loss (CAL) at six sites around the tooth on all teeth. All clinical parameters were measured with a Williams periodontal probe (Carl Martin). The PISA score was calculated from PPD, REC, and BOP, as suggested by Nesse at al. (2008) [4]. Clinical measurements were performed by an experienced periodontist (MKR). In the conducted study, intra-examiner calibration was performed on a sample of 10 patients with at least 20 teeth focusing on PPD measurement. The results demonstrated a high level of consistency, with a match rate of 93% for measurements within 1 mm or less. The clinical examination was supplemented by X-ray diagnostics. The study was approved by the National Medical Ethics Committee of the Republic of Slovenia (No-0120-602/2019/3 [6.2.2020]). The purpose of the study was explained to the patients who met the inclusion criteria, and they signed an informed consent form to participate in the study.

### 2.2. Blood Sample, Buccal Swab Collection, and DNA Extraction

A buccal swab was taken from the volunteers who agreed to participate in the study. The Isohelix Buccal-Prep Plus DNA Isolation Kit (Cell Projects Ltd., Kent, England, UK) was used to isolate the mtDNA and nDNA from the buccal swab. From the samples, the ratio between mitochondrial encoded MT-ND1 gene (mtDNA) and HBB gene encoded by nuclear DNA (nDNA) was determined by qPCR using the QuantStudio 12K Flex Real-Time PCR System, Applied Biosystems (Thermo Fisher Scientific, Waltham, MA, USA) according to the protocol of Shin et al. (2017) [46], and number of copies (mtDNAcn) was calculated according to the protocol of Quiros et al. (2018) [34]. Circulating cell-free DNA (ccfDNA) was extracted from 1 mL blood plasma using QIAamp ccfDNA/RNA Kit (Qiagen, Hilden, Germany). The plasma levels of mtDNA (ccf mtDNA) were assessed based on the MT-ND1 plasma levels, which were determined by absolute quantification using qPCR to assess copy number of mitochondrial gene MT-ND1 from blood plasma (ccf mtDNAcn).

All described measures were performed at the Centre for Human Molecular Genetics and Pharmacogenomics (CHMGF) at the Faculty of Medicine in Maribor, Slovenia. The subjects were referred to the laboratory of the Municipal Health Centre in Maribor for blood sampling, where the blood parameters were measured.

### 2.3. Statistical Analyses

We first compared the periodontal parameters of subjects whose values for the systemic parameters were within the physiological reference range with those who were above or below the reference values. In the second step, we compared the values of the systemic parameters of subjects with a high periodontal inflammatory burden (higher PISA value) with those with low values. Given the above-mentioned correlation between systemic and periodontal parameters and mitochondrial metabolism, in the final step, we attempted to establish a relationship between periodontal parameters, systemic inflammatory parameters, and mtDNAcn from buccal swabs.

The mtDNAcn was determined as the primary outcome. Due to the lack of previous data and the exploratory nature of the study, a detailed calculation of the exact number of patients to meet the statistical power of 0.8 was not possible. However, based on the data obtained, the number of patients meeting this requirement was calculated *post hoc* (see Results).

Categorical variables were described with frequencies and percentages, normally distributed continuous variables with means and standard deviations, and non-normally distributed variables with medians and interquartile ranges (IQR). The correlation between the PISA score and the continuous variables was examined using the Spearman correlation coefficient. The difference in the PISA score between two groups according to the reference values of the laboratory parameters was tested using the Mann–Whitney U test. Based on the PISA scores, the patients were divided into two groups—the bottom three quarters and the top quarter of patients. The association between the PISA groups and the laboratory and other parameters was examined by univariate logistic regression where possible, and by likelihood ratio or Mann–Whitney U test in other cases, as appropriate. Patients were also divided into two groups according to the presence of deep pockets. Patients without deep pockets (PPD ≥ 5 mm) served as controls. The association between demographic, laboratory, and other parameters and the presence of deep pockets was tested using a univariate logistic model. Program SPSS, v. 29 was used for statistical analysis. No correction for multiple testing was applied. All statistical tests were performed with a significance level of 0.05.

## 3. Results

Approximately 52 subjects were included in the study. A total of 22 (42.3%) were male. The mean (SD) age of the patients was 46.2 (8.8) years, and the mean (SD) BMI was 27.3 (4.1). Half of the patients exercised 2 h or fewer per week (IQR: 1–4), 1 patient finished primary school, 31 finished middle school, and the others finished faculty. All belonged to the middle socioeconomic class, and all had jobs; therefore, this information did not affect the research, and we did not include it in the statistics. The values of the laboratory tests, the number and percentage of patients with values of the laboratory tests within and outside the reference range, and qPCR data are shown in Table 1. In the analysed patient population, the most detected values outside the reference range were for cholesterol (87%), LDL (86%), SOD (80%), coenzyme Q (72%), and creatinine (76%).

Half of the patients had 54 or fewer sites with PPD ≥ 5 mm (IQR: 4–73). Half of the patients had 28 or fewer teeth (IQR: 27–30). Half of the patients had a PISA score of 14.5 or lower (IQR: 0.3–21.1). There is a strong correlation between the PISA score and the number of deep pockets per tooth (Spearman r = 0.95; *p* < 0.001).

There was a statistically significant correlation between the number of leukocytes, neutrophils, and CRP and the PISA value. The correlation is moderately strong (0.39–0.43). A significant marginal correlation was identified between PISA and NLR, uric acid, haemoglobin, ferritin, and calcium levels. No statistically significant correlation was found for other parameters examined. Both patients with a leukocyte count outside the reference range had a PISA value of 0, while half of the patients with a leukocyte count within the reference range had a PISA value of 15.76 or lower (IQR: 0.42–21.38). The PISA value was lower (Me [IQR]: 0.1 (0–8.3) in patients with uric acid values outside the reference range compared to patients with values within the range (Me [IQR]: 17.75 [7.91; 22.59]) (*p* < 0.001). Both patients with calcium outside the reference range had higher PISA scores (Me [IQR]: 29.44 [24.06–34.81]) compared to those with calcium within the reference range (Me [IQR]: 13.64 [0.31–20.73]) (*p* = 0.043). The relationship between the PISA score, demographic status, laboratory values, and values related to mitochondrial dysfunction, as well as the comparison of the PISA score between patients with parameters within the reference range and those with values outside the reference range, using the Mann–Whitney U test are shown in Table 2.

Table 3 summarizes the median (IQR) values of blood parameters by PISA groups after dividing the participants into two groups according to PISA score (in the second column are participants with PISA scores of lower than or equal to 21, and in the third column are those with PISA scores of more than 21) using univariate logistic regression (or the Mann–Whitney U test where indicated). The two groups differ in the number of erythrocytes. Patients with higher values of erythrocytes have ~5 times higher odds (95% CI: 1–24.9) for belonging to the top-quarter PISA group. Calcium values are higher in the top-quarter PISA group. Due to the high value of the odds ratio, the Mann–Whitney U test was used to test the difference in calcium values between the two PISA groups. The difference was statistically significant (*p* = 0.033). Patients with higher Mg/Ca values have close to zero odds (95% CI: 0–0.51) of belonging to the top-quarter PISA group. The amount of coenzyme Q10 is higher in the top-quarter PISA group. The odds of belonging to the top-quarter PISA group are ~14 times higher (95% CI: 1.98; 103.15) with each mmol/L increase in the amount of coenzyme Q10. Marginal significance was noted for the probabilities of being in the highest quartile of PISA values for Hb (OR 1.06 [1; 1.12]) and CRP (OR 1.19 [0.99; 1.42]). No association between other blood parameters and PISA groups was found.

Table 4 shows differences between participants with blood parameters outside the recommended range having a PISA score of lower than or equal to 21 and those with a score of higher than 21 using univariate logistic regression (or the Likelihood ratio test where indicated).

There were no patients with uric acid values outside the recommended range of 210–420 µmol/L in the top-quarter PISA group (Table 4). Eleven (28.2%) patients in the lower PISA group had uric acid values outside the recommended range. The difference is statistically significant (*p* = 0.007). No patients in the bottom PISA group had values of calcium outside the recommended range of 2.1–2.6 mmol/L, while there were 2 (15.4%) such patients in the top-quarter PISA group (*p* = 0.016). No statistically significant association between the values of other parameters according to their reference range and PISA group was found.

An association between mtDNAcn in buccal swabs and participants grouped by PISA scores (≤21 in the second column and >21 in the third column, using univariate logistic regression) was not observed. To further validate the results from buccal swab testing, we conducted additional experiments measuring circulating cell-free (ccf) mtDNAcn in blood plasma. Similarly, no association was detected between ccf mtDNAcn in blood plasma and PISA groupings (Table 5). Based on our pilot study, the calculated odds ratio between 2 × 2 delta and PISA >21 is 1.001. Detecting such an odds ratio as statistically significant at a 5% level with 80% power would require a sample size of 95 patients.

The patients were also divided according to the number of deep periodontal pockets with PPD ≥ 5 mm, the group with no deep pockets (n = 18), and the group with deep pockets (n = 34) (Table 6). The latter consists of individuals with 31 till 104 deep pockets. The comparison of the two groups showed that they statistically significantly differ in leukocyte count (*p* = 0.004)—the odds of having deep pockets are higher with a higher leucocyte count (OR [95% CI]: 2.14 [1.27–3.6]), neutrophil count (*p* = 0.005)—the odds of having deep pockets are higher with a higher neutrophil count (OR [95% CI]: 2.37 [1.36–5.49]), and uric acid values (*p* = 0.046)—the odds of having deep pockets are higher with higher uric acid values (OR [95% CI]: 1.01 [1–1.01]). A marginal significance was noted for the difference between groups regarding Hb (*p* = 0.083), CRP (*p* = 0.097), and NLR (*p* = 0.097).

## 4. Discussion

The aim of this study was to determine the association between periodontal parameters, systemic factors, and the number of mtDNA copies extracted from buccal swabs. Our analysis showed a moderately strong correlation between the PISA score and CRP and leukocyte count, especially neutrophils, after patients were categorized according to PISA score. It was also found that patients with deep periodontal pockets had an Increased number of leukocytes and neutrophils compared to healthy individuals, while the CRP value did not reach the threshold of statistical significance. The PISA values in the group of patients with uric acid values above or below the reference values were unexpectedly lower than the PISA values in subjects with uric acid values within the reference range. Corresponding to the number of deep pockets, patients with higher blood uric acid levels were more likely to have a higher number of deep periodontal pockets. Remarkably, the uric acid variable also showed statistical significance when included in the multivariate model. Patients with a higher erythrocyte count, a lower Mg/Ca ratio, and a higher blood CoQ10 level were more likely to belong to the group with the higher PISA score. We did not find statically significant differences in mtDNAcn between healthy individuals and patients with periodontal disease, regardless of whether patients were categorized by PISA score or the number of deep periodontal pockets.

A moderately strong positive correlation was found between the number of leukocytes and neutrophils in blood samples and the PISA score. When we grouped the subjects according to deep pockets, the likelihood of having deep pockets was higher with higher leukocyte and neutrophil counts. Another study confirmed a positive correlation between PISA and neutrophil count [48]. Similar results were also shown in a review and meta-analysis from 2021, in which severe chronic periodontitis was associated with a higher white blood cell count (MD = 0.53, CI: 0.26–0.79) and a higher neutrophil count (MD = 7.16%, 95% CI: 5.96–8.37). The number of leukocytes and neutrophils decreased after non-surgical periodontal therapy [49]. Periodontitis was also associated with a higher white blood cell count in the case-control study by Kumar et al. (2014) [50] and in the population of Japanese factory workers [42].

A moderately strong positive correlation was also found between CRP in blood samples and the PISA score. Other studies have shown similar results. Chronic and aggressive forms of periodontitis were associated with higher CRP levels in serum [9,43] and in saliva [51] than in healthy subjects, with the value for aggressive periodontitis being more than 50% higher than that for chronic periodontitis. A study by Kumari et al. (2024) showed a positive correlation between PISA and CRP. CRP levels decreased after non-surgical periodontal therapy (NSPT) [43]. After NSPT, CRP levels in saliva also decreased in non-smoking patients [10]. An increased white blood cell count, and an elevated CRP level are expected to be a response to periodontitis, which occurs as part of the inflammatory cascade [52].

After dividing the subjects into two groups according to PISA value, with 21 cm^2^ being the cut-off value, they differed in the number of erythrocytes (Er). Patients with a higher Er count are about 5 times more likely to belong to the top quartile of the PISA group. Many studies have come to different conclusions. Anand et al. (2014) showed a lower mean Er number in the group with generalized aggressive periodontitis compared to healthy subjects (4.45 ± 0.6 × 10^6^ vs. 4.78 ± 0.56 × 10^6^, *p* = 0.002) [5]. A study from Brazil showed that the number of circulating Er in the chronic periodontitis group was lower than in the healthy group (MD = −0.51, 95% CI: −0.78, −0.24, *p* = 0.0002) [13] and similarly in the study by Mann et al. (2017) [53]: (healthy [3.69–5.29 × 10^6^/µL] vs. periodontitis [3.33–5.97 × 10^6^/µL]). A reduced red blood cell count may be the result of gum bleeding, reduced longevity, proliferation and differentiation of red blood cells, or impaired iron metabolism [54]. It is also possible that a reduced number of Er is the cause of periodontitis because the oxygen supply to the periodontal tissues is impaired [5]. After periodontal treatment, there was a significant increase in the number of Er [14]. There are two studies that found a positive correlation between Er count and periodontitis, as in our study: one with localized periodontitis S II/III in military personnel from Taiwan [15], and the other with generalized severe periodontitis in young adults from the United States [55]. One possible reason for this is that all studies categorized patients into chronic and aggressive periodontitis; only our study and the study by Feng et al. (2022) categorized patients into groups according to the new periodontitis classification of 2017 [1]. As far as we know, our study is the only one that links PISA to the Er number.

Differences between the PISA groups were also found in blood calcium levels. In subjects with higher Mg/Ca levels, the probability of belonging to the top quarter of the PISA group is almost zero. Other studies confirm our findings. Meisel et al. (2016) [17] showed a dose–response relationship between Mg/Ca and CAL or tooth loss at 5-year follow-up. Patients in the lowest category of serum Mg/Ca had a higher prevalence of periodontitis, and those in the highest category had a lower mean pocket depth, attachment loss, and more teeth present compared to the group with the lowest Mg/Ca [16]. Yoshihara at al. (2011) [56] confirmed a dose–response relationship with periodontitis in smokers, but not in non-smokers.

When we compared subjects with blood parameters within and outside the reference range, there were significant differences in uric acid levels in the blood of subjects outside the reference range compared to those within the reference range. The PISA score was lower regardless of whether the score was below or above the reference range compared to those whose score was within the range. When subjects were divided into those without and those with deep pockets, subjects with higher blood uric acid levels were more likely to have deep pockets. The results of a systematic review and meta-analysis by Ye et al. (2023) [57] were similar: Significantly higher blood levels (WMD = 1.00 mg/dL, 95% CI 0.63 to 1.37, *p* < 0.001) and lower salivary levels (SMD = −1.57, 95% CI −2.25 to −0.90, *p* < 0.001) and GCF values (4.87 ± 0.36 vs. 5.11 ± 0.53 [mg/dL], *p* < 0.001) for uric acid were found in patients with periodontitis compared to healthy subjects. Differences were also found between severe and moderate to mild periodontitis. Similar results were found in a study from Taiwan, in which gout patients showed a higher risk of periodontitis, which decreased after treatment of the gout [58]. Higher blood uric acid levels could be due to accelerated purine degradation in periodontal tissues and systemic organs and/or increased secretion of uric acid from immune cells stimulated by periodontal pathogens [59,60]. Other studies have shown an antioxidant role of uric acid and a neuroprotective function [61]. The antioxidant effect has not yet been clearly demonstrated in periodontal diseases [57].

Significant differences were found in the amount of CoQ10, with the subjects with higher values belonging to the upper quarter of the PISA group. For each mmol/L increase in CoQ10, subjects were 14 times more likely to be in the top quartile of the PISA group. Most articles focus on the effect of CoQ10 in the treatment of periodontitis, as it is assumed that periodontitis leads to an increased formation of ROS and, thus, to an increased consumption of CoQ10, or that the progression of periodontitis is due to a reduced concentration of CoQ10 in the gingival tissue [23]. After the topical intra- or subgingival administration of exogenous CoQ10, periodontal parameters improved [25], and there was also a positive effect on oxidative stress markers, such as total antioxidant capacity, malondialdehyde, glutathione peroxidase, and catalase [62]. In a systematic review that included 17 studies since 2012, there was no clear evidence that CoQ10 has any additional benefit in non-surgical therapy [26]. Many patients have low CoQ10 levels, but it is possible that elevated CoQ10 levels in advanced periodontal disease are the result of an endogenous antioxidant trying to fight inflammation.

We found no correlation between the PISA score and the mtDNAcn or between the number of deep pockets and the mtDNAcn from the buccal swab. To our knowledge, no previously published studies have found a correlation between the PISA score and the mtDNAcn, and the buccal swab has not been used as a source of mtDNA and nDNA to determine the progression of periodontal disease, so it is difficult to compare our study with others. The rationale for using buccal swabs in the diagnosis of periodontitis lies in the non-invasive, cost-effective, and patient-oriented detection of biomarkers associated with the disease. Buccal swabs can be used to collect oral epithelial cells that provide valuable insights into the host’s immunological responses, which are often altered in periodontitis. mtDNAcn in blood has been correlated with various chronic diseases [63], such as cardiovascular disease, where inverse correlation was found [64,65,66], same as for liver disease [67,68], insulin resistance [69], Parkinson disease [70], some cancers, such as pancreatic [71], breast, and bladder cancer, and head and neck squamous cell cancer [72], but not, for example, in colorectal cancer [73] or lung adenocarcinoma [72]. mtDNAcn was also elevated in patients with end-stage renal disease [74] and with systemic scleroderma [75]. These differences in cancer patients could be due to a self-protective mechanism to prevent apoptosis [76] or a compensatory mechanism [77]. There are some studies showing an abnormal structure of mitochondria in gingival epithelial cells, gingival fibroblasts, and immune cells caused by *Porphyromonas gingivalis* [28,78] and mitochondrial mutation and dysfunction in periodontitis [29,79], but there are not many studies showing a link between periodontitis and mtDNAcn. For example, Zhou et al. (2024) did not demonstrate any causal relationship between mtDNAcn and periodontitis [27]. Sun et al. (2017) compared the mtDNAcn of four groups (controls, with periodontitis, with diabetes, and with both periodontitis and diabetes) of Wistar rats [80]. The mtDNAcn decreased significantly more in the diabetic and periodontitis groups (by 66%) and in the periodontitis group (by 40%) compared to healthy controls, but there was no significant difference between the controls and the rats with diabetes [80].

Blood is most commonly used to determine mtDNAcn, but any other tissue can be used [81]. Buccal swabs have been used as an effective alternative to traditional methods of tissue sampling for mtDNA and nDNA in animals and humans because they are non-invasive, rapid, and inexpensive [44,45,82,83,84]. However, buccal swabs can have some limitations. We need to be cautious in interpretation, as the buccal swab contains a larger proportion of epithelial cells and a smaller proportion of leukocytes, and the ratio between samples can vary greatly [85]. Theda et al. (2018) compared cellular heterogeneity in buccal swabs and saliva from adults and children and confirmed different proportions of epithelial cells and leukocytes [84]. Heterogeneity may influence the mtDNA/nDNA ratio, as epithelial cells have a different mtDNAcn content than leukocytes. For an accurate determination of the mtDNA/nDNA ratio, it is important to know the ratio of cells in the buccal swab [86], and in our study, the ratio of cells was not measured. On the other hand, buccal epithelial cells have a remarkably fast turnover rate, typically between 4 and 6 days [87] and 14–21 days in other literature [88]. This rapid renewal process means that the genetic material derived from these cells may not accurately reflect chronic conditions such as periodontitis, particularly if the disease state affects cellular properties over a prolonged period. Another potential problem is, while we have found a difference between healthy subjects and patients with stage III periodontitis, we do not know with certainty the rate of progression of periodontitis. Therefore, there is a possibility that compensatory mechanisms are also involved at certain rates of progression, leading to an increase in mtDNAcn to compensate for the deficient energy production. Due to the strict inclusion criteria and the expensive procedures for determining mtDNAcn and nDNAcn, the number of subjects in the study is low. On the other hand, the strict inclusion criteria and the relatively homogeneous group of subjects are strengths of the study. For this reason, it would be useful to conduct a multicentre study that would include more subjects with the same strict criteria as in our study, but who are included in the study over a longer period to also determine the progression rate and to perform more consecutive buccal swabs to find possible differences in the different progression rates.

The correlation between BMI, LDL, and HDL cholesterol and periodontitis indicates a complicated interplay between metabolic health and chronic inflammation. Increased BMI often correlates with increased LDL cholesterol and decreased HDL cholesterol, both of which are associated with systemic inflammation and oxidative stress. These metabolic changes can promote the progression of periodontitis, a chronic inflammation that affects the supporting tissue of the teeth. In addition, periodontitis can exacerbate metabolic dysfunction by increasing systemic inflammatory markers, potentially exacerbating lipid abnormalities. The association between obesity, lipid profiles, and periodontitis emphasise the need to maintain both systemic and oral health to mitigate the risk of associated problems [89]. However, these associations were not confirmed in our study, as participants were systematically healthy and randomly assigned BMI and/or cholesterol values outside the reference ranges.

## 5. Conclusions

A systemic inflammatory-immune response to periodontitis, as indicated by PISA and sites with PPD ≥ 5 mm, was characterized by fluctuations in blood CRP, leukocyte, neutrophil, erythrocyte, uric acid, and CoQ10 levels.

As a buccal swab is non-invasive and easy to handle, it can serve as an effective source of mtDNA and nDNA. Nevertheless, we found no correlation between the number of mtDNAcn from the buccal swab and the PISA score or number of deep pockets.

## Figures and Tables

**Table 1:** Values of blood parameters and qPCR data in patients.

Laboratory Test of Blood Parameters	Reference Range of Laboratory Parameters	Value of Laboratory Test		n and (%) of Participants (Out of 52) Within the Reference Range of Each Lab Parameter
Sedimentation	0–15 mm/h	Median (IQR):	9.5 (6–14.5)	42 (80.8)
Leucocytes	4–10 10^9^/L	Mean (SD):	6.3 (1.5)	50 (96.2)
Erythrocytes	4.5–5.5 10^12^/L	Mean (SD):	4.7 (0.4)	31 (59.6)
Hb	130–170 g/L	Mean (SD):	142 (12.5)	43 (82.7)
Platelets	50–410 10^9^/L	Mean (SD):	261 (59.6)	51 (98.1)
Neutrophils	1.5–7.4 10^9^/L	Mean (SD):	3.6 (1.2)	51 (98.1)
Lymphocytes	1.1–3.5 10^9^/L	Mean (SD):	2 (0.5)	50 (96.2)
NLR		Mean (SD):	1.96 (0.78)	
Fibrinogen	2–4 g/L	Median (IQR):	3 (2.6–3.5)	46 (88.5)
Glucose	3.6–6.1 mmol/L	Mean (SD):	5.7 (0.5)	41 (78.8)
HbA1c	4.3–6.1%	Median (IQR):	5.2 (5.1–5.3)	51 (98.1)
Bilirubin	<17 μmol/L	Median (IQR):	13 (9–19)	38 (73.1)
AST	<0.58 μkat/L	Median (IQR):	0.4 (0.3–0.5)	44 (84.6)
ALT	<0.74 μkat/L	Median (IQR):	0.4 (0.3–0.6)	43 (82.7)
Gamma GT	<0.92 μkat/L	Median (IQR):	0.4 (0.2–0.6)	45 (86.5)
Urea	2.8–7.5 mmol/L	Mean (SD):	5.3 (1.3)	50 (96.2)
Uric acid	210–420 mmol/L	Mean (SD):	304.9 (92.6)	41 (78.8)
Iron	10.7–28.6 μmol/L	Mean (SD):	18.6 (6)	46 (88.5)
Transferrin	2–3.8 g/L	Mean (SD):	2.7 (0.3)	50 (96.2)
Ferritin	20–300 μg/L	Median (IQR):	79.5 (39.5–134.5)	48 (92.3)
Triglycerides	0.6–1.7 mmol/L	Median (IQR):	1.04 (0.75–1.41)	43 (82.7)
HDL	>1 mmol/L	Mean (SD):	1.42 (0.36)	48 (92.3)
Cholesterol	4–5 mmol/L	Mean (SD):	5.42 (0.91)	13 (25)
LDL	<3 mmol/L	Mean (SD):	3.49 (0.84)	14 (26.9)
CRP	<5 mg/L	Median (IQR):	2.1 (0.9–3.9)	44 (84.6)
Ca	2.10–2.60 mmol/L	Median (IQR):	2.43 (2.34–2.5)	50 (96.2)
Mg	0.6–1.10 mmol/L	Mean (SD):	0.88 (0.07)	52 (100)
Mg/Ca		Median (IQR):	0.36 (0.344–0.382)	
Lactate	0.5–2.2 mmol/L	Median (IQR):	1.2 (0.9–1.8)	46 (88.5)
Creatinine	0.3–0.7 mg/dL	Mean (SD):	0.7 (0.21)	24 (46.2)
CoQ10	0.8–1.4 mmol/L	Mean (SD):	1.15 (0.42)	28 (53.8)
SOD	164–240 U/mL	Median (IQR):	246 (205.5–284)	20 (38.5)
qPCR data	Me (IQR)			
Ct MT-ND1	12.7 (12.1–13.3)	SD MT-ND1	0 (0–0.1)	
Ct HBB	20.6 (20.1–22.8)	SD HBB	0.1 (0–0.1)	
deltaCt	8.4 (7.8–9.3)			
mtDNAcn	694.2 (460.2–1265.9)			

Values are presented as mean and standard deviation or median and interquartile range for laboratory tests and as n (numbers) or % out of a total 52. Abbreviations include haemoglobin (Hb), neutrophils and lymphocytes ratio (NLR), glycated haemoglobin (Hb1Ac), alanine aminotransferase (ALT), aspartate aminotransferase (AST), gamma-glutamyl transferase (gamma GT), high-density lipoprotein cholesterol (HDL), low-density lipoprotein cholesterol (LDL), C-reactive protein (CRP), calcium (Ca), magnesium (Mg), magnesium and calcium ratio (Mg/Ca), Coenzyme Q10 (CoQ10), superoxide dismutase (SOD), cycle threshold of mitochondrial DNA encoded nicotinamide adenine dinucleotide dehydrogenase 1 gene (Ct MT-ND1), cycle threshold of nuclear DNA encoded haemoglobin beta gene (Ct HBB), difference between Ct-MT-ND1 and Ct HBB (delta Ct), and mitochondrial DNA copy number (mtDNAcn). The Ct value is a critical measure in qPCR, representing the cycle number at which the fluorescence generated by the PCR product crosses a defined threshold level. This threshold is set during the exponential phase of amplification, where the amount of DNA doubles with each cycle. A lower Ct value indicates a higher initial quantity of target nucleic acid, while a higher Ct suggests a lower initial quantity [47].

**Table 2:** Correlation between demographic status, blood parameters (hemogram and biochemistry tests), mitochondrial DNA copy number, and PISA score, using Spearman correlation coefficient (*p*-value) (second column); comparison of PISA value (median (IQR); n) between patients having a parameter within the reference range and those with values outside the range; and results of Mann–Whitney U test (third, fourth, and fifth columns).

		Parameters’ Reference Values		
Demographic Status, Laboratory Tests, Buccal Swab mtDNAcn	PISA Value Spearman Correlation (*p*)	Laboratory Tests Within Reference Range PISA (Me (IQR)); n	Laboratory Tests Outside Reference Range PISA (Me (IQR)); n	*p* (Between Within and Outside the Reference Range)
Age	−0.05 (0.73)			
BMI	0.2 (0.152)			
Sports activity	0.08 (0.571)			
Sedimentation	0.11 (0.452)	11.1 (0.23–20.45); 42	19.24 (13.49–24.06); 10	0.125
Leukocytes	**0.42 (0.002)**	15.76 (0.42–21.38); 50	0 (0–0); 2	**0.028**
Erythrocytes	0.24 (0.087)	15.23 (0.5–22.59); 31	13.49 (0.04–20.73); 21	0.327
Hb	*0.27 (0.053)*	13.79 (0.42–21.38); 43	17.52 (0–20.82); 9	0.553
Platelets	−0.05 (0.728)	15.23 (0.31–21.38); 51	11.39 (11.39–11.39); 1	0.868
Neutrophils	**0.43 (0.002)**	15.23 (0.33–21.38); 51	0 (0–0); 1	0.125
Lymphocytes	0.11 (0.424)	15.76 (0.31–21.38); 50	4.12 (0.33–7.91); 2	0.341
NLR	*0.27 (0.052)*			
Fibrinogen	0.17 (0.231)	12.44 (0.23–20.45); 46	22.4 (17.75–24.98); 6	0.053
Glucose	0.09 (0.525)	13.49 (0.23–20.82); 41	17.22 (7.91–24.98); 11	0.370
Hba1c	−0.02 (0.878)	15.23 (0.33–21.38); 51	0 (0–0); 1	0.125
Bilirubin	−0.12 (0.406)	17.37 (0.5–21.42); 38	4.24 (0.15–17.08); 14	0.890
AST	0.02 (0.879)	15.76 (0.28–20.78); 44	10.41 (4.17–23.33); 8	0.919
ALT	0.07 (0.649)	15.23 (0.23–20.73); 43	11.39 (8.03–24.06); 9	0.594
Gamma GT	0.09 (0.536)	13.79 (0.31–20.45); 45	20.82 (8.03–24.06); 7	0.295
Urea	0.1 (0.468)	14.51 (0.33–20.82); 50	10.69 (0–21.38); 2	0.651
Uric acid	*0.27 (0.054)*	17.75 (7.91–22.59); 41	0.1 (0–8.03); 11	**<0.001**
Iron	−0.06 (0.696)	14.51 (0.31–20.82); 46	12.1 (0.33–22.59); 6	1
Transferrin	0.03 (0.834)	14.51 (0.31–20.82); 50	16.04 (10.65–21.42); 2	0.634
Ferritin	*0.25 (0.077)*	15.76 (0.37–21.4); 48	0.45 (0.17–9.15); 4	0.175
Triglycerides	0.03 (0.845)	16.28 (0.42–20.82); 43	9.42 (0–22.59); 9	0.208
HDL	0 (0.985)	15.76 (0.32–21.1); 48	10.76 (4.01–18.77); 4	0.655
Cholesterol	0.22 (0.124)	17.22 (10.65–20.45); 13	13.49 (0.15–21.42); 39	0.612
LDL	0.18 (0.2)	17.37 (0.42–21.42); 14	12.44 (0.31–20.82); 38	0.606
CRP	**0.39 (0.004)**	11.1 (0.19–20.64); 44	20.18 (15.66–22.74); 8	0.091
Ca	*0.24 (0.086)*	13.64 (0.31–20.73); 50	29.44 (24.06–34.81); 2	**0.043**
Mg	−0.07 (0.62)	14.51 (0.32–21.1); 52	-	-
Mg/Ca	−0.19 (0.174)			
Lactate	0.02 (0.911)	13.64 (0.31–21.38); 46	18.24 (10.81–19.62); 6	0.557
Creatin	−0.08 (0.58)	16.68 (0.24–19.67); 24	12.59 (0.37–21.4); 28	0.993
CoQ10	0.26 (0.067)	11.1 (0.27–20.04); 28	17.49 (0.38–23.33); 24	0.291
SOD	0.02 (0.912)	17.63 (0.1–20.78); 20	11.1 (0.38–21.4); 32	0.836
mtDNAcn	0.05 (0.711)			

Body mass index (BMI), sports activity (number of hours per week), haemoglobin (Hb), neutrophils and lymphocytes ratio (NLR), glycated haemoglobin (Hb1Ac), alanine aminotransferase (ALT), aspartate aminotransferase (AST), gamma-glutamyl transferase (gamma GT), high-density lipoprotein cholesterol (HDL), low-density lipoprotein cholesterol (LDL), C-reactive protein (CRP), calcium (Ca), magnesium (Mg), magnesium and calcium ratio (Mg/Ca), Coenzyme Q10 (CoQ10), superoxide dismutase (SOD),mitochondrial DNA copy number (mtDNAcn).

**Table 3:** Association between blood parameters and PISA groups (univariate logistic regression or Mann–Whitney U test where indicated).

	PISA < 21 (n = 39) Me (IQR)	PISA > 21 (n = 13) Me (IQR)	OR (95% CI)	*p*
Sedimentation	9 (6; 14)	11 (5; 16)	1.02 (0.93; 1.11)	0.73
Leukocytes	6.1 (4.9; 7)	6.7 (5.9; 7.8)	1.34 (0.87; 2.07)	0.181
Erythrocytes	4.6 (4.4; 5)	4.9 (4.6; 5.3)	4.99 (1; 24.91)	**0.05**
Hb	140 (133; 147)	147 (140; 156)	1.06 (1; 1.12)	*0.054*
Platelets	269 (217; 310)	250 (206; 259)	0.99 (0.98; 1)	0.25
Neutrophils	3.4 (2.5; 4.4)	3.8 (3; 4.7)	1.4 (0.82; 2.37)	0.215
Lymphocytes	2 (1.5; 2.3)	2 (1.7; 2.2)	1.44 (0.43; 4.8)	0.551
NLR	1.9 (1.2; 2.5)	2.1 (1.7; 2.4)	1.23 (0.55; 2.75)	0.609
Fibrinogen	3 (2.6; 3.5)	3.1 (2.5; 3.2)	1.32 (0.63; 2.76)	0.456
Glucose	5.7 (5.5; 6)	5.6 (5.3; 6)	0.82 (0.2; 3.41)	0.786
Hba1c	5.2 (5.1; 5.3)	5.2 (5; 5.3)	0.94 (0.11; 8.04)	0.956
Bilirubin	13 (9; 20)	11 (7; 17)	0.97 (0.89; 1.06)	0.494
AST	0.37 (0.32; 0.43)	0.46 (0.37; 0.52)	5.29 (0.12; 243.28)	0.394
ALT	0.38 (0.28; 0.56)	0.51 (0.4; 0.75)	1.73 (0.36; 8.3)	0.491
Gama GT	0.35 (0.23; 0.57)	0.28 (0.24; 0.73)	1.8 (0.47; 6.9)	0.389
Urea	5.1 (4.1; 6.5)	5 (4.8; 5.9)	1.22 (0.74; 2.01)	0.433
Uric acid	288 (221; 379)	322 (302; 382)	1 (1; 1.01)	0.216
Iron	18.6 (14.7; 21.6)	16.6 (13; 21.1)	0.99 (0.89; 1.1)	0.796
Transferrin	2.7 (2.5; 2.8)	2.7 (2.4; 3.1)	1.81 (0.25; 12.8)	0.554
Ferritin	69 (38; 121)	112 (82; 213)	1 (1; 1.01)	0.22
Triglycerides	1 (0.8; 1.4)	1.1 (0.7; 1.5)	1.81 (0.67; 4.88)	0.24
HDL	1.4 (1.1; 1.7)	1.3 (1.2; 1.7)	1.03 (0.18; 5.96)	0.974
Cholesterol	5.2 (4.8; 6)	5.5 (5.3; 6.3)	1.73 (0.83; 3.57)	0.141
LDL	3.3 (2.9; 3.7)	3.9 (3; 4.2)	1.73 (0.79; 3.79)	0.171
CRP	1.9 (0.9; 3.5)	3.5 (1.5; 4.2)	1.19 (0.99; 1.42)	*0.06*
Ca	2.41 (2.32; 2.47)	2.51 (2.38; 2.54)	-	**0.033 ^a^**
Mg	0.88 (0.84; 0.92)	0.86 (0.82; 0.9)	0.03 (0; 498.47)	0.47
Mg/Ca	0.4 (0.3; 0.4)	0.3 (0.3; 0.4)	0 (0; 0.51)	**0.043**
Lactate	1.2 (0.9; 1.7)	1 (0.8; 2)	0.83 (0.31; 2.22)	0.712
Creatin	0.7 (0.5; 0.9)	0.7 (0.6; 0.9)	0.66 (0.03; 13.99)	0.787
Coenzyme Q10	1 (0.8; 1.2)	1.5 (1.4; 1.7)	14.3 (1.98; 103.15)	**0.008**
SOD	245 (217; 278)	271 (179; 326)	1 (1; 1.01)	0.352

Haemoglobin (Hb), neutrophils and lymphocytes ratio (NLR), glycated haemoglobin (Hb1Ac), alanine aminotransferase (ALT), aspartate aminotransferase (AST), gamma-glutamyl transferase (gamma GT), high-density lipoprotein cholesterol (HDL), low-density lipoprotein cholesterol (LDL), C-reactive protein (CRP), calcium (Ca), magnesium (Mg), magnesium and calcium ratio (Mg/Ca), Coenzyme Q10 (CoQ10), superoxide dismutase (SOD). ^a^ Mann–Whitney U test.

**Table 4:** Association between reference values of blood parameters and PISA groups (univariate logistic regression or Likelihood ratio test where indicated).

Out of Recommended Range:	PISA < 21	PISA > 21	OR (95% CI)	*p*
	Me (IQR)	Me (IQR)		
Sedimentation	6 (15.4)	4 (30.8)	2.44 (0.57; 10.57)	0.232
Leukocytes	2 (5.1)	0 (0)	-	0.278 ^a^
Erythrocytes	17 (43.6)	4 (30.8)	0.58 (0.15; 2.19)	0.417
Hb	7 (17.9)	2 (15.4)	0.83 (0.15; 4.62)	0.833
Platelets	1 (2.6)	0 (0)	-	0.446 ^a^
Neutrophils	1 (2.6)	0 (0)	-	0.446 ^a^
Lymphocytes	2 (5.1)	0 (0)	-	0.278 ^a^
Fibrinogen	3 (7.7)	3 (23.1)	3.6 (0.63; 20.65)	0.151
Glucose	8 (20.5)	3 (23.1)	1.16 (0.26; 5.24)	0.845
Hba1c	1 (2.6)	0 (0)	-	0.446 ^a^
Bilirubin	12 (30.8)	2 (15.4)	0.41 (0.08; 2.14)	0.289
AST	5 (12.8)	3 (23.1)	2.04 (0.41; 10.06)	0.381
ALT	5 (12.8)	4 (30.8)	3.02 (0.67; 13.63)	0.15
Gama GT	4 (10.3)	3 (23.1)	2.62 (0.5; 13.72)	0.253
Urea	1 (2.6)	1 (7.7)	3.17 (0.18; 54.57)	0.427
Uric acid	11 (28.2)	0 (0)	-	**0.007 ^a^**
Iron	4 (10.3)	2 (15.4)	1.59 (0.26; 9.89)	0.619
Transferrin	1 (2.6)	1 (7.7)	3.17 (0.18; 54.57)	0.427
Ferritin	4 (10.3)	0 (0)	-	0.548 ^a^
Triglycerides	6 (15.4)	3 (23.1)	1.65 (0.35; 7.82)	0.528
HDL	3 (7.7)	1 (7.7)	1 (0.09; 10.54)	1
Cholesterol	28 (71.8)	11 (84.6)	2.16 (0.41; 11.37)	0.363
LDL	29 (74.4)	9 (69.2)	0.78 (0.2; 3.08)	0.718
CRP	5 (12.8)	3 (23.1)	2.04 (0.41; 10.06)	0.381
Ca	0 (0)	2 (15.4)	-	**0.016 ^a^**
Mg	0 (0)	0 (0)	-	-
Lactate	5 (12.8)	1 (7.7)	0.57 (0.06; 5.35)	0.62
Creatin	20 (51.3)	8 (61.5)	1.52 (0.42; 5.48)	0.522
Coenzyme Q10	16 (41)	8 (61.5)	2.3 (0.64; 8.33)	0.205
SOD	23 (59)	9 (69.2)	1.57 (0.41; 5.97)	0.512

Haemoglobin (Hb), neutrophils and lymphocytes ratio (NLR), glycated haemoglobin (Hb1Ac), alanine aminotransferase (ALT), aspartate aminotransferase (AST), gamma-glutamyl transferase (gamma GT), high-density lipoprotein cholesterol (HDL), low-density lipoprotein cholesterol (LDL), C-reactive protein (CRP), calcium (Ca), magnesium (Mg), magnesium and calcium ratio (Mg/Ca), Coenzyme Q10 (CoQ10), superoxide dismutase (SOD). ^a^ Likelihood ratio test.

**Table 5:** Association between mtDNAcn from buccal swab or ccf mtDNAcn from blood plasma and PISA groups.

	PISA < 21	PISA > 21		
	Me (IQR)	Me (IQR)	OR (95% CI)	*p*
mtDNAcn from buccal swab	618.1 (328; 1191.9)	846.3 (675.9; 1337.7)	1 (1; 1)	0.174
ccf mtDNAcn from blood plasma	62,604.3 (30,902.1; 126,482.7)	43,927.9 (25,161.5; 78,252.1)	1 (1; 1)	0.674

mtDNAcn—copy number of mitochondrial gene MT-ND1 isolated from buccal swab. ccf mtDNAcn—circulating cell free (ccf) DNA copy number of mitochondrial gene MT-ND1 from blood plasma.

**Table 6:** Association between patients’ demographic characteristics, blood parameters, and mtDNAcn divided according to number of deep periodontal pockets (data shown as means [SD] or median [IQR] if not stated differently).

	Pockets ≥ 5 mm			
	No (n = 18) Mean (SD) or Me (IQR)	Yes (n = 34) Mean (SD) or Me (IQR)	OR (95% CI)	*p*
Male; n (%)	6 (33.3)	16 (47.1)	1.78 (0.54; 5.84)	0.343
Age	46.2 (7.9)	46.1 (9.4)	1 (0.94; 1.07)	0.994
BMI	25.5 (23.8–28.7)	26.9 (25.2–30.4)	1.07 (0.92; 1.23)	0.377
Sports activity (h/week)	3 (1–4)	2 (1–4)	0.99 (0.77; 1.28)	0.945
Sedimentation	9 (7–12)	11 (5–15)	1 (0.92; 1.08)	0.934
Leukocytes	5.4 (1.5)	6.7 (1.3)	2.14 (1.27; 3.6)	**0.004**
Erythrocytes	4.6 (0.5)	4.7 (0.4)	2.15 (0.51; 9.09)	0.298
Hb	137.8 (12.6)	144.3 (12.1)	1.05 (0.99; 1.1)	*0.083*
Platelets	258 (217–316)	257.5 (213–277)	1 (0.99; 1.01)	0.941
Neutrophils	2.6 (2.1–3.4)	3.8 (3.1–4.7)	2.73 (1.36; 5.49)	**0.005**
Lymphocytes	1.8 (0.5)	2 (0.5)	2.27 (0.7; 7.33)	0.17
NLR	1.71 (0.86)	2.09 (0.71)	2.04 (0.88; 4.73)	*0.097*
Fibrinogen	2.8 (2.3–3.4)	3.1 (2.6–3.5)	1.39 (0.65; 2.94)	0.394
Glucose	5.6 (5.2–5.8)	5.7 (5.5–6.1)	2.97 (0.72; 12.31)	0.134
Hba1c	5.2 (5.1–5.3)	5.2 (5–5.3)	2.03 (0.29; 14.36)	0.479
Bilirubin	12.5 (9–22)	13 (8–17)	0.99 (0.92; 1.06)	0.773
AST	0.4 (0.4–0.4)	0.4 (0.3–0.5)	0.97 (0.02; 39.43)	0.987
ALT	0.4 (0.3–0.5)	0.4 (0.3–0.6)	1.71 (0.32; 9.31)	0.532
Gama GT	0.3 (0.2–0.5)	0.4 (0.2–0.6)	2.17 (0.43; 10.91)	0.347
Urea	5.2 (1.3)	5.4 (1.3)	1.12 (0.71; 1.77)	0.613
Uric acid	268.6 (117)	324.2 (71.4)	1.01 (1; 1.01)	**0.046**
Iron	17.8 (15.9–20.9)	18.75 (14–21.7)	1.04 (0.94; 1.15)	0.463
Transferrin	2.7 (0.3)	2.7 (0.4)	1.17 (0.21; 6.54)	0.861
Ferritin	51 (38–77)	102.5 (48–157)	1.01 (1; 1.01)	0.212
Triglycerides	1.13 (0.71–1.53)	1.01 (0.75–1.38)	1.1 (0.42; 2.9)	0.844
HDL	1.46 (0.39)	1.41 (0.35)	0.67 (0.13; 3.44)	0.636
Cholesterol	5.25 (1.07)	5.51 (0.82)	1.37 (0.71; 2.65)	0.342
LDL	3.3 (0.95)	3.59 (0.78)	1.55 (0.74; 3.23)	0.244
CRP	1.1 (0.5–2.9)	2.8 (1.3–4)	1.3 (0.95; 1.78)	*0.097*
Ca	2.43 (2.32–2.47)	2.42 (2.34–2.51)	7.58 (0.06; 957.09)	0.412
Mg	0.88 (0.06)	0.88 (0.07)	0.57 (0; 3194.74)	0.897
Mg/Ca	0.36 (0.35–0.38)	0.36 (0.34–0.38)	0 (0; 29,518.71)	0.478
Lactate	1.2 (0.9–1.3)	1.3 (0.9–2)	2.01 (0.72; 5.58)	0.181
Creatin	0.7 (0.2)	0.69 (0.23)	0.47 (0.03; 7.85)	0.597
Coenzyme Q10	1.13 (0.75–1.33)	1.1 (0.9–1.5)	1.08 (0.27; 4.3)	0.911
SOD	243 (218–272)	260 (202–285)	1 (0.99; 1.01)	0.678
mtDNAcn	698.6 (482.1–1323.7)	689.9 (446.3–1191.9)	1 (1; 1)	0.721

Body mass index (BMI), sports activity (number of hours per week), haemoglobin (Hb), neutrophils and lymphocytes ratio (NLR), glycated haemoglobin (Hb1Ac), alanine aminotransferase (ALT), aspartate aminotransferase (AST), gamma-glutamyl transferase (gamma GT), high-density lipoprotein cholesterol (HDL), low-density lipoprotein cholesterol (LDL), C-reactive protein (CRP), calcium (Ca), magnesium (Mg), magnesium and calcium ratio (Mg/Ca), Coenzyme Q10 (CoQ10), superoxide dismutase (SOD), mitochondrial DNA copy number (mtDNAcn).

## Data Availability

The raw data supporting the conclusions of this article will be made available by the authors on request.

## References

[1] Papapanou P.N., Sanz M., Buduneli N., Dietrich T., Feres M., Fine D.H., Flemmig T.F., Garcia R., Giannobile W.V., Graziani F. (2018). Periodontitis: Consensus Report of Workgroup 2 of the 2017 World Workshop on the Classification of Periodontal and Peri-Implant Diseases and Conditions. J. Clin. Periodontol..

[2] Lim G., Janu U., Chiou L.L., Gandhi K.K., Palomo L., John V. (2020). Periodontal Health and Systemic Conditions. Dent. J..

[3] Nomura Y., Morozumi T., Numabe Y., Ogata Y., Nakayama Y., Sugaya T., Nakamura T., Sato S., Takashiba S., Sekino S. (2021). Estimation of the Periodontal Inflamed Surface Area by Simple Oral Examination. J. Clin. Med..

[4] Nesse W., Abbas F., Van Der Ploeg I., Spijkervet F.K.L., Dijkstra P.U., Vissink A. (2008). Periodontal Inflamed Surface Area: Quantifying Inflammatory Burden. J. Clin. Periodontol..

[5] Anand P.S., Sagar D.K., Ashok S., Kamath K.P. (2014). Association of Aggressive Periodontitis with Reduced Erythrocyte Counts and Reduced Hemoglobin Levels. J. Periodontal Res..

[6] Loos B.G., Craandijk J., Hoek F.J., Dillen P.M.E.W., Velden U. (2000). Van Der Elevation of Systemic Markers Related to Cardiovascular Diseases in the Peripheral Blood of Periodontitis Patients. J. Periodontol..

[7] Acharya A.B., Shetty I.P., Jain S., Padakannaya I., Acharya S., Shettar L., Thakur S. (2019). Neutrophil-to-Lymphocyte Ratio and Platelet-to-Lymphocyte Ratio in Chronic Periodontitis before and after Nonsurgical Therapy. J. Indian. Soc. Periodontol..

[8] Almășan O., Leucuța D.C., Hedeșiu M. (2022). Blood Cell Count Inflammatory Markers as Prognostic Indicators of Periodontitis: A Systematic Review and Meta-Analysis. J. Pers. Med..

[9] Stathopoulou P.G., Buduneli N., Kinane D.F. (2015). Systemic Biomarkers for Periodontitis. Curr. Oral Health Rep..

[10] Hadžić Z., Puhar I. (2021). C-Reactive Protein in Saliva of Non-Smoking Patients with Periodontitis (a Pilot Study). J. Health Sci..

[11] Hajishengallis G., Chavakis T. (2021). Local and Systemic Mechanisms Linking Periodontal Disease and Inflammatory Comorbidities. Nat. Rev. Immunol..

[12] Neupane S.P., Virtej A., Myhren L.E., Bull V.H. (2022). Biomarkers Common for Inflammatory Periodontal Disease and Depression: A Systematic Review. Brain Behav. Immun. Health.

[13] de Carvalho França L.F., da Silva F.R.P., di Lenardo D., Alves E.H.P., Nascimento H.M.S., da Silva I.A.T., Vasconcelos A.C.C.G., Vasconcelos D.F.P. (2019). Comparative Analysis of Blood Parameters of the Erythrocyte Lineage between Patients with Chronic Periodontitis and Healthy Patients: Results Obtained from a Meta-Analysis. Arch. Oral Biol..

[14] Jain K., Jyoti Das S., Dwivedi S., Jain R., Nugala B., Jain M. (2016). Effect of Periodontal Treatment on Red Blood Cell Parameters in Patients with Chronic Periodontitis. Int. J. Dent. Res. Dev..

[15] Feng A.C., Tsai S.C., Lin Y.P., Tsai K.Z., Lin G.M. (2022). Erythrocyte Indices and Localized Stage II/III Periodontitis in Military Young Men and Women: CHIEF Oral Health Study. BMC Oral Health.

[16] Merchant A.T. (2006). Higher Serum Magnesium:Calcium Ratio May Lower Periodontitis Risk. J. Evid.-Based Dent. Pract..

[17] Meisel P., Pink C., Nauck M., Jablonowski L., Voelzke H., Kocher T. (2016). Magnesium/Calcium Ratio in Serum Predicts Periodontitis and Tooth Loss in a 5-Year Follow-Up. JDR Clin. Trans. Res..

[18] Patil R.T., Dhadse P.V., Salian S.S., Punse S.D. (2024). Role of Oxidative Stress in Periodontal Diseases. Cureus.

[19] Bansal D.N., Gupta D.N.D. (2014). Role Of Dietary Antioxidants In Periodontitis: A Preventive Approach. IOSR J. Dent. Med. Sci..

[20] Byun S.H., Yoo D.M., Lee J.W., Choi H.G. (2020). Analyzing the Association between Hyperuricemia and Periodontitis: A Cross-Sectional Study Using Koges Hexa Data. Int. J. Environ. Res. Public Health.

[21] Xu J., Jia Y., Mao Z., Wei X., Qiu T., Hu M. (2023). Association between Serum Uric Acid, Hyperuricemia and Periodontitis: A Cross-Sectional Study Using NHANES Data. BMC Oral Health.

[22] Yubero D., Allen G., Artuch R., Montero R. (2017). The Value of Coenzyme Q10 Determination in Mitochondrial Patients. J. Clin. Med..

[23] Prakash S., Sunitha J., Hans M. (2010). Role of Coenzyme Q 10 as an Antioxidant and Bioenergizer in Periodontal Diseases. Indian J. Pharmacol..

[24] Barakat A.K., Attia A. (2019). Clinical Evaluation of Co-Enzyme Q10 in Management of Chronic Periodontitis Patients: Mouth Split Study. Int. J. Health Sci. Res..

[25] Kadir A., Rabbi A.A., Rahman M.M. (2017). CoEnzyme Q10: A New Horizon in the Treatment of Periodontal Diseases. Int. Dent. J. Stud. Res..

[26] Merle C.L., Lenzen C., Schmalz G., Ziebolz D. (2023). Systematic Review on Protocols of Coenzyme Q10 Supplementation in Non-Surgical Periodontitis Therapy. Nutrients.

[27] Zhou H., Qi Y.X., Cao R.Y., Zhang X.X., Li A., Pei D.D. (2024). Causal Relationship between Mitochondrial Biological Function and Periodontitis: Evidence from a Mendelian Randomization Study. Int. J. Mol. Sci..

[28] Liu J., Wang Y., Shi Q., Wang X., Zou P., Zheng M., Luan Q. (2022). Mitochondrial DNA Efflux Maintained in Gingival Fibroblasts of Patients with Periodontitis through ROS/MPTP Pathway. Oxid. Med. Cell. Longev..

[29] Kannan B., Arumugam P. (2023). The Implication of Mitochondrial DNA Mutation and Dysfunction in Periodontal Diseases. J. Indian. Soc. Periodontol..

[30] Deng Y., Xiao J., Ma L., Wang C., Wang X., Huang X., Cao Z. (2024). Mitochondrial Dysfunction in Periodontitis and Associated Systemic Diseases: Implications for Pathomechanisms and Therapeutic Strategies. Int. J. Mol. Sci..

[31] Habbane M., Montoya J., Rhouda T., Sbaoui Y., Radallah D., Emperador S. (2021). Human Mitochondrial DNA: Particularities and Diseases. Biomedicines.

[32] Furtwängler A., Reiter E., Neumann G.U., Siebke I., Steuri N., Hafner A., Lösch S., Anthes N., Schuenemann V.J., Krause J. (2018). Ratio of Mitochondrial to Nuclear DNA Affects Contamination Estimates in Ancient DNA Analysis. Sci. Rep..

[33] Carpagnano G.E., Lacedonia D., Malerba M., Palmiotti G.A., Cotugno G., Carone M., Foschino-Barbaro M.P. (2016). Analysis of Mitochondrial DNA Alteration in New Phenotype ACOS. BMC Pulm. Med..

[34] Quiros P.M., Goyal A., Jha P., Auwerx J. (2017). Analysis of MtDNA/NDNA Ratio in Mice. Curr. Protoc. Mouse Biol..

[35] Al-Kafaji G., Aljadaan A., Kamal A., Bakhiet M. (2018). Peripheral Blood Mitochondrial DNA Copy Number as a Novel Potential Biomarker for Diabetic Nephropathy in Type 2 Diabetes Patients. Exp. Ther. Med..

[36] Bordoni L., Petracci I., Pelikant-Malecka I., Radulska A., Piangerelli M., Samulak J.J., Lewicki L., Kalinowski L., Gabbian-elli R., Olek R.A. (2021). Mitochondrial DNA copy number and trimethylamine levels in the blood: New insights on cardio-vascular disease biomarkers. FASEB J..

[37] Zheng J., Ninghua C., Zhang S., Xuebin W., Ming L. (2019). Leukocyte Mitochondrial DNA Copy Number and Risk of Thyroid Cancer: A Two-Stage Case-Control Study. Front. Endocrinol..

[38] Wang D., Li Z., Liu W., Zhou J., Ma X., Tang J., Chen X. (2018). Differential Mitochondrial DNA Copy Number in Three Mood States of Bipolar Disorder. BMC Psychiatry.

[39] Randeu H., Bronkhorst A.J., Mayer Z., Oberhofer A., Polatoglou E., Heinemann V., Haas M., Boeck S., Holdenrieder S. (2022). Preanalytical Variables in the Analysis of Mitochondrial DNA in Whole Blood and Plasma from Pancreatic Cancer Patients. Diagnostics.

[40] Carpagnano G.E., Lacedonia D., Cotugno G., Malerba M., Patricelli G. (2017). Changes of Mitochondria Copy Number in Association with Idiopathic Pulmonary Fibrosis. Clin. Res. Pulmonol..

[41] Zamora-Perez A.L., Zúñiga-González G.M., Gómez-Meda B.C., Lazalde-Ramos B.P., Ortiz-García Y.M., Morales-Velazquez G., Velázquez C.G., Sánchez-Parada M.G. (2017). Periodontal Disease and Nuclear and Oxidative DNA Damage. Insights into Various Aspects of Oral Health.

[42] Inoue K., Kobayashi Y., Hanamura H., Toyokawa S. (2005). Association of Periodontitis with Increased White Blood Cell Count and Blood Pressure. Blood Press..

[43] Machado V., Botelho J., Escalda C., Hussain S.B., Luthra S., Mascarenhas P., Orlandi M., Mendes J.J., D’Aiuto F. (2021). Serum C-Reactive Protein and Periodontitis: A Systematic Review and Meta-Analysis. Front. Immunol..

[44] Maggo S., North L.Y., Ozuna A., Ostrow D., Grajeda Y.R., Hakimjavadi H., Cotter J.A., Judkins A.R., Levitt P., Gai X. (2024). A Method for Measuring Mitochondrial DNA Copy Number in Pediatric Populations. Front. Pediatr..

[45] Solanky D., Fields J.A., Iudicello J.E., Ellis R.J., Franklin D., Clifford D.B., Gelman B.B., Marra C.M., Morgello S., Rubin L.H. (2022). Higher Buccal Mitochondrial DNA and Mitochondrial Common Deletion Number Are Associated with Markers of Neurodegeneration and Inflammation in Cerebrospinal Fluid. J. Neurovirol..

[46] Shin J., Kim K.C., Lee D.C., Lee H.R., Shim J.Y. (2017). Association between Salivary Mitochondrial DNA Copy Number and Chronic Fatigue According to Combined Symptoms in Korean Adults. Korean J. Fam. Med..

[47] Adams G. (2020). A Beginner’s Guide to RT-PCR, QPCR and RT-QPCR. Biochemist.

[48] Kumari R., Banerjee A., Verma A., Kumar A., Biswas N., Kumari P. (2024). Assessing the Correlation of Periodontal Inflamed Surface Area (PISA) With Systemic Inflammatory Markers. Cureus.

[49] Botelho J., Machado V., Hussain S.B., Zehra S.A., Proença L., Orlandi M., Mendes J.J., D’Aiuto F. (2021). Periodontitis and Circulating Blood Cell Profiles: A Systematic Review and Meta-Analysis. Exp. Hematol..

[50] Kumar B.-P., Khaitan T., Ramaswamy P., Sreenivasulu P., Uday G., Velugubantla R.-G. (2014). Association of Chronic Periodontitis with White Blood Cell and Platelet Count-A Case Control Study. J. Clin. Exp. Dent..

[51] Almoharib H.S., Almubarak A., Alrowis R., Geevarghese A., Preethanath R.S., Anil S. (2014). Conflict of Interest: None Source of Support: Nil Oral Fluid Based Biomarkers in Periodontal Disease: Part 1. Saliva. J. Int. Oral Health.

[52] Bhattacharya H., Srivastava R., Gummaluri S., Agarwal M., Bhattacharya P., Astekar M. (2022). Comparison of Blood Parameters between Periodontitis Patients and Healthy Participants: A Cross-Sectional Hematological Study. J. Oral Maxillofac. Pathol..

[53] Mann V., Subramanyam M., Verma R., Jha A., John J.R. (2017). Estimation and Comparison of Erythrocyte and Hemoglobin Levels in Subjects with Healthy Periodontium and Chronic Periodontitis. Pesqui. Bras. Odontopediatria Clin. Integr..

[54] Muñoz M., Villar I., García-Erce J.A. (2009). An Update on Iron Physiology. World J. Gastroenterol..

[55] Hu M., Zhang W., Shi Z., Dai B., Liu X., Cheng X., Zhang H., Wang Y. (2022). Association Between Hematological Parameters and Generalized Severe Periodontitis Among Young Adults in the United States. Res. Sq..

[56] Yoshihara A., Iwasaki M., Miyazaki H. (2011). Mineral Content of Calcium and Magnesium in the Serum and Longitudinal Periodontal Progression in Japanese Elderly Smokers. J. Clin. Periodontol..

[57] Ye L., Zhao L., Mei Z., Zhou Y., Yu T. (2023). Association between Periodontitis and Uric Acid Levels in Blood and Oral Fluids: A Systematic Review and Meta-Analysis. BMC Oral Health.

[58] Chen H.H., Ho C.W., Hsieh M.C., Chen C.C., Hsu S.P., Lin C.L., Kao C.H. (2020). Gout Can Increase the Risk of Periodontal Disease in Taiwan. Postgrad. Med..

[59] Jun H.K., Jung Y.J., Choi B.K. (2017). Treponema Denticola, *Porphyromonas gingivalis*, and Tannerella Forsythia Induce Cell Death and Release of Endogenous Danger Signals. Arch. Oral Biol..

[60] Jun H.K., An S.J., Kim H.Y., Choi B.K. (2020). Inflammatory Response of Uric Acid Produced by *Porphyromonas gingivalis* Gingipains. Mol. Oral Microbiol..

[61] Hou W., Xia X., Li Y., Lv H., Liu J., Li X. (2022). Recent Progress and Perspectives on the Relationship between Hyperuricemia and Periodontitis. Front. Immunol..

[62] Sangsefidi Z.S., Yaghoubi F., Hajiahmadi S., Hosseinzadeh M. (2020). The Effect of Coenzyme Q10 Supplementation on Oxidative Stress: A Systematic Review and Meta-analysis of Randomized Controlled Clinical Trials. Food Sci. Nutr..

[63] Castellani C.A., Longchamps R.J., Sun J., Guallar E., Arking D.E. (2020). Thinking Outside the Nucleus: Mitochondrial DNA Copy Number in Health and Disease. Mitochondrion.

[64] Ashar F.N., Zhang Y., Longchamps R.J., Lane J., Moes A., Grove M.L., Mychaleckyj J.C., Taylor K.D., Coresh J., Rotter J.I. (2017). Association of Mitochondrial DNA Copy Number with Cardiovascular Disease. JAMA Cardiol..

[65] Tin A., Grams M.E., Ashar F.N., Lane J.A., Rosenberg A.Z., Grove M.L., Boerwinkle E., Selvin E., Coresh J., Pankratz N. (2016). Association between Mitochondrial DNA Copy Number in Peripheral Blood and Incident CKD in the Atherosclerosis Risk in Communities Study. J. Am. Soc. Nephrol..

[66] Zhang Y., Guallar E., Ashar F.N., Longchamps R.J., Castellani C.A., Lane J., Grove M.L., Coresh J., Sotoodehnia N., Ilkhanoff L. (2017). Association between Mitochondrial DNA Copy Number and Sudden Cardiac Death: Findings from the Atherosclerosis Risk in Communities Study (ARIC). Eur. Heart J..

[67] Pirola C.J., Scian R., Gianotti T.F., Dopazo H., Rohr C., Martino J.S., Castaño G.O., Sookoian S. (2015). Epigenetic Modifications in the Biology of Nonalcoholic Fatty Liver Disease: The Role of DNA Hydroxymethylation and TET Proteins. Medicine.

[68] Yuan Y., Ju Y.S., Kim Y., Li J., Wang Y., Yoon C.J., Yang Y., Martincorena I., Creighton C.J., Weinstein J.N. (2020). Comprehensive Molecular Characterization of Mitochondrial Genomes in Human Cancers. Nat. Genet..

[69] Sookoian S., Rosselli M.S., Gemma C., Burgueño A.L., Fernández Gianotti T., Castaño G.O., Pirola C.J. (2010). Epigenetic Regulation of Insulin Resistance in Nonalcoholic Fatty Liver Disease: Impact of Liver Methylation of the Peroxisome Proliferator-Activated Receptor γ Coactivator 1α Promoter. Hepatology.

[70] Pyle A., Anugrha H., Kurzawa-Akanbi M., Yarnall A., Burn D., Hudson G. (2015). Reduced Mitochondrial DNA Copy Number Is a Biomarker of Parkinson’s Disease. Neurobiol. Aging.

[71] Lynch S.M., Weinstein S.J., Virtamo J., Lan Q., Liu C.S., Cheng W.L., Rothman N., Albanes D., Stolzenberg-Solomon R.Z. (2011). Mitochondrial DNA Copy Number and Pancreatic Cancer in the Alpha-Tocopherol Beta-Carotene Cancer Prevention Study. Cancer Prev. Res..

[72] Reznik E., Miller M.L., Şenbabaoğlu Y., Riaz N., Sarungbam J., Tickoo S.K., Al-Ahmadie H.A., Lee W., Seshan V.E., Hakimi A.A. (2016). Mitochondrial DNA Copy Number Variation across Human Cancers. Elife.

[73] Thyagarajan B., Wang R., Barcelo H., Koh W.P., Yuan J.M. (2012). Mitochondrial Copy Number Is Associated with Colorectal Cancer Risk. Cancer Epidemiol. Biomark. Prev..

[74] Eirin A., Saad A., Tang H., Herrmann S.M., Woollard J.R., Lerman A., Textor S.C., Lerman L.O. (2016). Urinary Mitochondrial DNA Copy Number Identifies Chronic Renal Injury in Hypertensive Patients. Hypertension.

[75] Bogatyreva A.I., Gerasimova E.V., Kirichenko T.V., Markina Y.V., Tolstik T.V., Kiseleva D.G., Popkova T.V., Markin A.M. (2023). Mitochondrial DNA Copy Number in Patients with Systemic Sclerosis. Front. Mol. Biosci..

[76] Mei H., Sun S., Bai Y., Chen Y., Chai R., Li H. (2015). Reduced MtDNA Copy Number Increases the Sensitivity of Tumor Cells to Chemotherapeutic Drugs. Cell Death Dis..

[77] Gasparre G., Romeo G., Rugolo M., Porcelli A.M. (2011). Learning from Oncocytic Tumors: Why Choose Inefficient Mitochondria?. Biochim. Biophys. Acta Bioenerg..

[78] Luo S., Xu T., Zheng Q., Jiang A., Zhao J., Ying Y., Liu N., Pan Y., Zhang D. (2024). Mitochondria: An Emerging Unavoidable Link in the Pathogenesis of Periodontitis Caused by *Porphyromonas gingivalis*. Int. J. Mol. Sci..

[79] Dong Z., Wu L., Hong H. (2023). Mitochondrial Dysfunction in the Pathogenesis and Treatment of Oral Inflammatory Diseases. Int. J. Mol. Sci..

[80] Sun X., Mao Y., Dai P., Li X., Gu W., Wang H., Wu G., Ma J., Huang S. (2017). Mitochondrial Dysfunction Is Involved in the Aggravation of Periodontitis by Diabetes. J. Clin. Periodontol..

[81] Hurtado-Roca Y., Ledesma M., Gonzalez-Lazaro M., Moreno-Loshuertos R., Fernandez-Silva P., Enriquez J.A., Laclaustra M. (2016). Adjusting MtDNA Quantification in Whole Blood for Peripheral Blood Platelet and Leukocyte Counts. PLoS ONE.

[82] Bordoni L., Smerilli V., Nasuti C., Gabbianelli R. (2019). Mitochondrial DNA Methylation and Copy Number Predict Body Composition in a Young Female Population. J. Transl. Med..

[83] Koutsokali M., Dianni C., Valahas M. (2023). Buccal Swabs as an Effective Alternative to Traditional Tissue Sampling Methods for DNA Analyses in Chamaeleonidae. Wildl. Biol..

[84] Theda C., Hwang S.H., Czajko A., Loke Y.J., Leong P., Craig J.M. (2018). Quantitation of the Cellular Content of Saliva and Buccal Swab Samples. Sci. Rep..

[85] Eipel M., Mayer F., Arent T., Ferreira M.R.P., Birkhofer C., Gerstenmaier U., Costa I.G., Ritz-Timme S., Wagner W. (2016). Epigenetic Age Predictions Based on Buccal Swabs Are More Precise in Combination with Cell Type-Specific DNA Methylation Signatures. Aging.

[86] Wong Y.T., Tayeb M.A., Stone T.C., Lovat L.B., Teschendorff A.E., Iwasiow R., Craig J.M. (2022). A Comparison of Epithelial Cell Content of Oral Samples Estimated Using Cytology and DNA Methylation. Epigenetics.

[87] Borthakur G., Butryee C., Stacewicz-Sapuntzakis M., Bowen P.E. (2008). Exfoliated Buccal Mucosa Cells as a Source of DNA to Study Oxidative Stress. Cancer Epidemiol. Biomark. Prev..

[88] Wang S.-S., Tang Y.-L., Pang X., Zheng M., Tang Y.-J., Liang X.-H. (2019). The Maintenance of an Oral Epithelial Barrier. Life Sci..

[89] Techatanawat S., Komchornrit A. (2021). Association of Lipid Profile and Body Mass Index with Periodontal Status in Patients with Dyslipidemia with and without Lipid-Lowering Medication: A Cross-Sectional Study. Oral Health Prev. Dent..

